# Endoscopic Transsphenoidal Surgery with a Layered Peel Strategy for Cushing’s Disease Treatment: A Case Series

**DOI:** 10.3390/brainsci13040671

**Published:** 2023-04-17

**Authors:** Chuan Shao, Junwei Wang, Pan Wang, Nan Wu

**Affiliations:** 1 Department of Neurosurgery, Chongqing General Hospital, Chongqing 401147, China; 2 Graduate Institute, Chongqing Medical University, Chongqing 400016, China

**Keywords:** Cushing’s disease, endoscopic transsphenoidal surgery, outcomes, pituitary adenoma

## Abstract

Patients with Cushing’s disease (CD) who underwent endoscopic transsphenoidal surgery (ETS) with a layered peel strategy at our center were retrospectively analyzed. Records on patients’ basic characteristics, preoperative and early postoperative evaluations, perioperative complications, and follow-up were collected. A total of 12 unselected, consecutive patients with CD were identified. Ten of the twelve patients were female. All tumors were confirmed by magnetic resonance imaging, with a maximum tumor diameter ranging from 5 mm to 11 mm. A lower rate of surgical complications was identified postoperatively, with no cases of carotid artery injury, epistaxis, hematoma, visual disturbance, or intracranial infection, but with one case of cerebrospinal fluid rhinorrhea. Ten patients experienced immediate remission, and two had delayed remission. No recurrence events were observed during a median follow-up of 51 months. In conclusion, our preliminary experience indicated that ETS with a layered peel strategy provided a perfect remission rate, low complication rate, and no recurrence in a case series of CD patients. Given the limited number of cases, future studies are warranted to confirm its effectiveness and safety.

## 1. Introduction

Cushing’s syndrome (CS) is a rare but life-endangering endocrine disorder characterized by excessive cortisol production [1]. Approximately 70% of CS cases are cases of Cushing’s disease (CD) caused by an adrenocorticotropin hormone (ACTH)-secreting pituitary tumor [2]. It is estimated that the annual incidence of CD is 1.2–1.7 individuals per million people [1]. CD is usually associated with multiple co-morbidities, such as diabetes mellitus, insulin resistance, obesity, dyslipidemia, immune suppression, hypertension, atherosclerosis, hypercoagulability, osteoporosis, and increased mortality risk, especially for infection and cardiovascular diseases [1,2]. Therefore, effective therapy to decrease the devastating burden of excess cortisol is essential. Transsphenoidal pituitary surgery is the first-line treatment for most patients with CD, although various therapies, including drugs, radiation therapy, and bilateral adrenalectomy, are available. As endoscopic technology has developed rapidly, endoscopic endonasal transsphenoidal procedures for CD have become more accessible. However, the previous literature has indicated that the outcomes after endoscopic transsphenoidal surgery (ETS) remain unsatisfactory, with a pooled recurrence rate of 11% (range: 0–23.1%) [3]. Purely endoscopic pituitary surgery has been adopted at our center since 2014. The present study aimed to investigate the clinical characteristics, treatment, and outcomes of 12 CD cases treated with ETS with a layered peel strategy by a pituitary neurosurgeon with extensive experience at a single institution.

## 2. Materials and Methods

Patients. A total of 12 consecutive patients with CD underwent ETS by a senior author (NW) who had performed about 1500 pituitary adenoma resection procedures between January 2014 and September 2022. Preoperative and early postoperative evaluations, data on complications during the operation or within the first few weeks following surgery, and follow-up records at outpatient doors were retrospectively reviewed.

Perioperative evaluation and management. The CD cases were diagnosed based on typical clinical hallmarks, elevated urinary free cortisol (UFC) excretion over the past 24 h, disruption of the circadian rhythm of cortisol secretion, a normal or increased plasma concentration of ACTH, the failure of overnight cortisol suppression tests after 1 mg dexamethasone administration or a low-dose two-day dexamethasone test, the success of overnight cortisol suppression via 8 mg dexamethasone administration or a high-dose two-day dexamethasone test, and pituitary lesion features detected on high-quality magnetic resonance imaging (MRI). Tumor size was defined based on the maximum tumor diameter, and tumors were divided into two categories of micro-adenomas (<10 mm) and macroadenomas (≥10 mm). Inferior petrosal sinus sampling (IPSS) was conducted if pituitary origin could not be determined. Moreover, additional thoracic and abdominal computed tomography (CT) or color Doppler ultrasound scans were performed to exclude an ectopic ACTH tumor. No perioperative steroids were administered for early remission assessment.

Surgical technique. The surgical strategy was based on the preoperative MRI results and biochemical assessment. Nan Wu carried out the endoscopic endonasal one-nostril approach without removing the middle turbinate. The following key principles were followed during the operation: (1) an attempt was made to remove all adenomas; (2) a layered peel strategy was adopted during the operation rather than blind curettage or suction; based on the authors’ experience, four important layers, including the pituitary capsule, pituitary gland, tumor, and pseudocapsule, were involved in the operation; (3) the tumor was removed en bloc if circumscription between pseudocapsule and normal pituitary gland could be identified and sufficient operating space was available; piecemeal tumor resection and subsequent complete pseudocapsule removal was performed in cases of limited operation space; and (4) partial hypophysectomy adjacent to the tumor was performed if a clear identification of circumscription could not be achieved. All lesions were histopathologically examined and immunohistochemically stained for pituitary hormones to ascertain the diagnosis of ACTH-secreting pituitary adenoma.

Postoperative management and follow-up. All patients received an MRI examination to determine whether the tumor was successfully removed within two days after surgery. Routine tests to determine the levels of serum ACTH, cortisol, and other hormones associated with thyrotropic, gonadotropic, and somatotropic axes were performed at 8:00 am on the first, third, fifth, and seventh days after surgery. Random cortisol level evaluations were performed, and cortisol replacement treatment was initiated until the HPA axis was restored when patients exhibited signs or symptoms of hypocortisolemia. Immediate remission was defined as a morning serum cortisol level of <138 nmol/L (5 µg/dL) or UFC level of <28–56 nmol/day (10–20 μg/dL) within 7 days after surgery [4,5]. Because a progressive decline in cortisol level to a normal or low level within three months after surgery has been identified in previous studies [6,7,8], patients without any additional therapy had a delayed remission with a low or normal morning serum cortisol level with a resolution of clinical features during the first three months of the follow-up period after resection. A recurrence of CD was defined as relapsing hypercortisolism or clinical deterioration occurring three months after surgery. Follow-up endocrinological and MRI examinations continued at 3, 6, and 12 months and then annually thereafter. All adverse events requiring further management within 30 days of the ETS were considered surgical complications. The adverse events included carotid artery injury, epistaxis, diabetes insipidus (DI), cerebrospinal fluid (CSF) rhinorrhea, hematoma, visual disturbance, and intracranial infection. The current study did not consider general complications, such as thromboembolic events, cardiovascular disease, pulmonary infections, and others.

## 3. Results

A total of 12 patients were diagnosed with CD between 2014 and 2022. Two of the twelve patients were male, and ten were female. All patients were adults with a mean age of 32.58 ± 10.81 years (range: 18–50 years). The median lag time from the presentation of clinical symptoms to CD diagnosis was 9.5 months (range: 3–84 months). Clinical features at the time of presentation are summarized in Table 1. All patients experienced weight gain and central obesity. Most patients exhibited facial swelling, round face, purple skin striae, thin skin, acne, hypertension, and hyperpigmentation.

A total of eight patients visited the dermatology department at our or another medical center and received some medical therapy before surgery. However, detailed drug information was not available. MRI examinations resulted in definitive abnormal images with microadenomas in all patients (range: 5–9 mm), except one with a maximum tumor diameter of 11 mm. None of the patients received an IPSS test. Moreover, nephrolithiasis (33.33%) was identified by CT scans and/or color Doppler ultrasound examinations.

**Figure 1:**
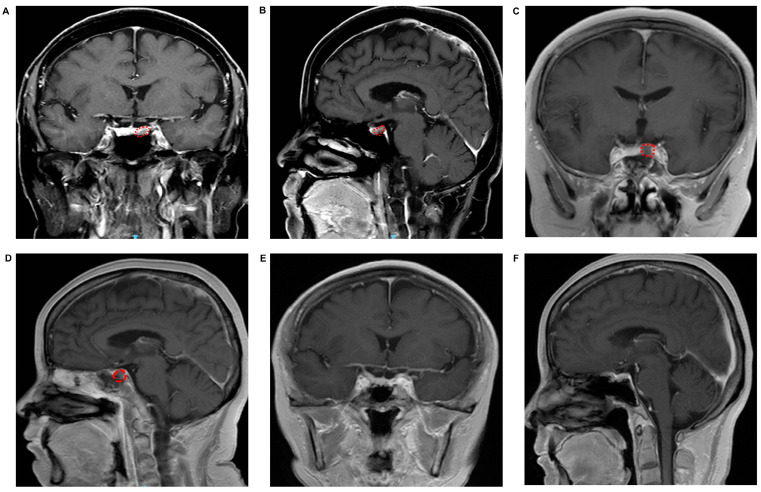
Preoperative and postoperative MRI. (**A**,**B**) Coronal and Sagittal MRI scans show a microadenoma adjacent to the left cavernous sinus. (**C**,**D**) Coronal and Sagittal MRI scans taken 2 days after surgery, indicating that the tumor was removed completely. (**E**,**F**) Coronal and Sagittal MRI scans taken 3 years after surgery, indicating no recurrence.

**Figure 2:**
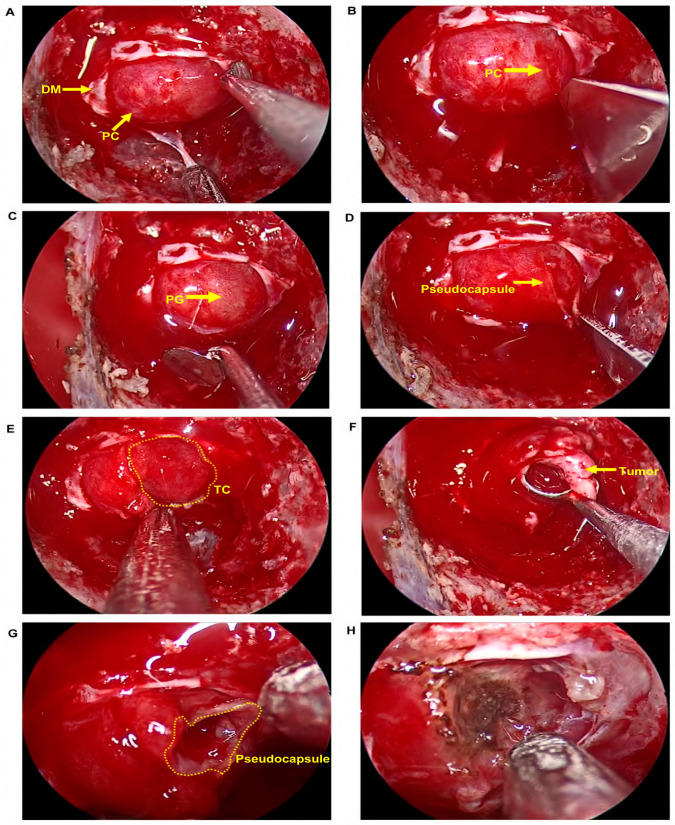
Endoscopic resection of CD. (**A**) The dura was opened without entering the pituitary capsule after the sellar base bone was removed. (**B**) The pituitary capsular was opened in a linear incision. (**C**) Blunting separation to expose the normal pituitary gland. (**D**,**E**) The pituitary gland was bluntly separated until tumor was fully exposed. (**F**) A pure piecemeal intratumoral resection. (**G**) Pseudocapsule. (**H**) The pseudocapsule was dissected thoroughly. Abbreviations: CD, Cushing’s disease; DM, dura mater; PC, pituitary capsule; PG, pituitary gland; TC, tumor circumscription.

Of the twelve patients, four underwent en bloc resection, while eight had a piecemeal intratumoral resection followed by a thorough pseudocapsule dissection. The present report described a representative case using some perioperative, intraoperative, and postoperative images and a surgery video. Figure 1A,B show a microadenoma adjacent to the left cavernous sinus before surgery. Figure 1C,D show the MRI scans on the second day after surgery, indicating that the tumor was resected. Figure 1E,F represent the re-examination MRI scans that were obtained at a three-year follow-up, showing no evidence of tumor recurrence. The ETS process is described in the surgery video (Video S1) and Figure 2. Briefly, the surgeon stood on the patient’s right side, and the endoscope was placed in the right nasal cavity. The middle turbinate and bony nasal septum were left intact during the operation. The sphenoid sinus opening was located under endoscopic guidance, the mucosa was cut, and the anterior wall of the sphenoid sinus was removed with high-speed grinding drills to expose the sellar floor. The dura was opened after removing the sellar base bone without entering the pituitary capsule (Figure 2A). Next, the location closest to the tumor was chosen based on the MRI results to open the pituitary capsule using a linear incision (Figure 2B) and to expose the normal pituitary gland via blunted separation (Figure 2C). Then, the pituitary gland was bluntly separated until the tumor was fully exposed (Figure 2D,E). A piecemeal intratumoral resection was performed due to limited space (Figure 2F), and the pseudocapsule was kept intact during the operation (Figure 2G) and subsequently thoroughly dissected (Figure 2H). Finally, artificial dura mater, Gelfoam, and SURGICEL FIBRILLAR were used to repair the skull base after thorough hemostasis followed by nasal packing with cotton pieces (Video S1).

Next, an MRI examination within 2 postoperative days confirmed the complete removal of the tumor. Immediate remission was achieved in 10 patients, and two delayed remissions were recorded during the subsequent three months of follow-up. Concerning the postoperative complications (Table 2), two patients experienced transient DI, one had short-term hypopituitarism and received prednisone replacement therapy, and one experienced CSF rhinorrhea and was successfully treated with lumbar subarachnoid drainage. Surgery did not induce carotid artery injury, epistaxis, hematoma, visual disturbance, or intracranial infection. All cases were identified by positive ACTH immunostaining. There were no recurrences following a median follow-up of 51 months (range: 7–99 months).

## 4. Discussion

The present study retrospectively reviewed 12 consecutive and newly diagnosed pa-tients with MRI-documented CD. Among these patients, 11 had microadenomas and 1 had macroadenoma (11 mm). All patients underwent ETS. After surgery, 10 patients experienced immediate remission and 2 had delayed remission. None of the patients experienced tumor recurrence at the last follow-up.

Reports on the CD remission rate after ETS vary at different medical centers, with re-mission rates around being 60–95% [3,9,10,11]. In addition to different study designs (sample size and follow-up time), tumor characteristics (invasiveness, tumor size, and localization), surgical experience, and adjuvant therapies, this may be due to the lack of a uniform consensus for the definition of remission, including immediate and delayed remission [12,13]. For example, a low (<50 nmol/L, 100 nmol/L, or 138 nmol/L) or normal morning serum cortisol level within 3, 4, or 7 days after surgery, clinical symptom disappearance, normal or decreased UFC levels over the past 24 h, the success of a low-dose dexamethasone test, or a combination of two or more aforementioned criteria was used to define tumor remission [4,11,14,15,16,17]. The criteria for CD recurrence seemed to be relatively consistent, including an increase in serum cortisol level, an increase in night salivary cortisol level, and/or an increase in 24 h UFC level, with clinical hallmarks reviewed by Qiao [3]. Therefore, establishing a good consensus statement regarding CD remission and recurrence after tumor resection is of the utmost importance for making a meaningful comparison of surgical outcomes in future studies.

The present series had a low rate of postoperative surgical complications, with no cases of carotid artery injury, epistaxis, hematoma, visual disturbance, or intracranial infection, but with one case of CSF rhinorrhea (8.3%). Other case series have reported an incidence rate ranging from 0.0% to 16.7% [18,19,20,21,22,23]. Endoscopic surgery theoretically offers better visualization of the tumor, particularly for large or laterally invasive tumors [24]. Thus, a more aggressive surgery may be adopted in an effort to remove the entire adenoma, which may lead to a higher risk of a CSF leak [24,25,26]. Epistaxis is a dangerous complication during and after surgery. Ligating the sphenopalatine and anterior ethmoidal artery is an excellent way to control epistaxis. However, anatomical variations of the anterior ethmoidal artery may occasionally be present. Recently, the three-dimensional reconstruction of CT angiography for the intraoperative localization and preoperatory assessment of variations of the anterior ethmoidal artery variations have been indicated, thus providing some insights into epistaxis treatment [27].

Since the pseudocapsule of pituitary tumors was introduced in 1936, remarkable progress in learning about its occurrence, development, histopathology, morphology, and clinical significance has been made [28,29,30,31,32,33,34]. The pseudocapsule-based extracapsular resection technique has been valued clinically by neurosurgeons because the method helps to improve the tumor resection rate, lower the recurrence rate, and protect the pituitary function. The surgeon in the present report adopted a layered peel strategy rather than blind curettage or suction during the operation. A location closest to the tumor was also chosen during surgery in order to open the pituitary capsule, thus preventing excessive damage to the normal pituitary gland. As indicated in the surgery video and Figure 2, the core of the strategy involved opening the pituitary capsule, partially incising it, and bluntly separating the pituitary gland before touching the tumor. Thus, these procedures can help to easily identify a clear circumscription between the tumor and the pituitary gland and keep the pseudocapsule of pituitary tumors intact. Consequently, the residual tumor could be minimized, thus improving the total tumor resection rate. As expected, the circumscription between the pseudocapsule and normal pituitary gland was clearly identified in all cases, and no patients underwent a partial hypophysectomy adjacent to the tumor.

Furthermore, there was a concern about hypopituitarism associated with this strategy. However, only one patient underwent short-term prednisone replacement therapy. Therefore, concerns about the safety of ETS with a layered peel strategy could be, at least partially, eliminated. Sometimes, the pituitary tissue is thin and fragile, and it is a real challenge for neurosurgeons to peel the partial pituitary gland successfully without inflicting more damage to it. Therefore, ETS with a layered peel strategy should be per-formed by an experienced, meticulous, and trained surgeon at centers with a high volume of neurosurgical cases.

Significantly, all patients with CD experienced remission in the present case series, and no tumor recurrence was detected during the follow-up. In addition to the merits of a layered peel strategy listed above, several limitations accounting for these findings should be noted. First, only 12 patients were included in the series. Thus, the study findings may be chance results. Second, due to a median follow-up of 51 months (range: 7–99 months), the durability of ETS needs to be confirmed during a lifelong follow-up period. Third, all tumors with a maximum tumor diameter of ≤11 mm and without an invasive phenotype were well-documented via MRI examinations. Notably, over 90% of CD cases were microadenomas, and few presented with an invasive phenotype [35]. However, approximately 30–50% of CD patients undergoing transsphenoidal surgery still required additional treatment, including pituitary radiotherapy, reoperation, adrenal surgery, and/or other medical therapy [36]. Given the poor state of CD treatment, the present surgical strategy provided some vital insights into CD surgery.

## 5. Conclusions

In conclusion, our preliminary experience indicated that ETS with a layered peel strategy provided a perfect remission rate, low complication rate, and no recurrence in a case series of CD patients. Given the limited number of cases, future studies are warranted to confirm its effectiveness and safety.

## Figures and Tables

**Table 1:** Basic characteristics.

Characteristics	Mean (SD) (Min–Max) or N (%)
Age (year)	32.58 (10.81) (18.00–50.00)
Hypertension	8 (66.67%)
Diabetes	3 (25.00%)
Dyslipidemia	2 (16.67%)
Weight gain	12 (100.00%)
Central obesity	12 (100.00%)
Headache	2 (16.67%)
Round face	10 (83.33%)
Backache	1 (8.33%)
Fatigue	1 (8.33%)
Hair loss	2 (16.67%)
Hyperpigmentation	8 (66.67%)
Skin purple striae	10 (83.33%)
Thin skin	9 (75.00%)
Hirsutism (women)	4 (40.00%)
Infertility (women)	1 (10%)
Menstrual irregularities (women)	5 (50.00%)
Acne	9 (75.00%)
Sleep disorders	2 (16.67%)
Nephrolithiasis	4 (33.33%)
Depression	1 (8.33%)
Osteoporosis	2 (16.67%)

Abbreviations: N, number.

**Table 2:** Postoperative complications of the patients who underwent ETS.

Complications	N (%)
Transient DI	2 (16.67%)
Hypopituitarism	1 (8.33%)
CSF rhinorrhea	1 (8.33%)
Carotid artery injury	0 (0%)
Epistaxis	0 (0%)
Hematoma	0 (0%)
Visual disturbance	0 (0%)
Intracranial infection	0 (0%)

Abbreviations: N, number.

## Data Availability

Data are available from the corresponding author upon reasonable request.

## Supplementary Materials

The following supporting information can be downloaded at: https://www.mdpi.com/article/10.3390/brainsci13040671/s1, Video S1: The major procedure of endoscopic transsphenoidal surgery.
